# From Tissue Physoxia to Cancer Hypoxia, Cost-Effective Methods to Study Tissue-Specific O_2_ Levels in Cellular Biology

**DOI:** 10.3390/ijms23105633

**Published:** 2022-05-18

**Authors:** Carlos H. V. Nascimento-Filho, Alexandra T. Glinos, Yeejin Jang, Eny M. Goloni-Bertollo, Rogerio M. Castilho, Cristiane H. Squarize

**Affiliations:** 1 Laboratory of Epithelial Biology, Department of Periodontics and Oral Medicine, University of Michigan School of Dentistry, Ann Arbor, MI 48109-1078, USA; carlosviesi@gmail.com (C.H.V.N.-F.); glinosat@umich.edu (A.T.G.); steljang@umich.edu (Y.J.); csquariz@umich.edu (C.H.S.); 2 Genetics and Molecular Biology Research Unit (UPGEM), Department of Molecular Biology, School of Medicine of São José do Rio Preto, São José do Rio Preto 15090-000, SP, Brazil; eny.goloni@famerp.br; 3 Rogel Cancer Center, University of Michigan, Ann Arbor, MI 48109-0944, USA

**Keywords:** hypoxia chamber, hypoxia, epithelial-mesenchymal transition, cancer stem cell

## Abstract

The human body is endowed with an extraordinary ability to maintain different oxygen levels in various tissues and organs. The maintenance of physiological levels of oxygen is known as physoxia. The development of hypoxic conditions plays an important role in the biology of several pathologies, including cancer. In vitro studies using normal and neoplastic cells require that culture conditions be carried out under appropriate oxygen levels, either physoxic or hypoxic conditions. Such requirements are difficult to widely implement in laboratory practice, mainly due to the high costs of specialized equipment. In this work, we present and characterize a cost-effective method to culture cells under a range of oxygen levels using deoxidizing pouches. Our results show that physoxic and hypoxic levels using deoxidizing absorbers can be achieved either by implementing a gradual change in oxygen levels or by a regimen of acute depletion of oxygen. This approach triggers the activation of an epithelial-mesenchymal transition in cancer cells while stimulating the expression of HIF-1α. Culturing cancer cells with deoxidizing agent pouches revealed PI3K oncogenic pathway exacerbations compared to tumor cells growing under atmospheric levels of oxygen. Similar to the PI3K signaling disturbance, we also observed augmented oxidative stress and superoxide levels and increased cell cycle arrest. Most interestingly, the culture of cancer cells under hypoxia resulted in the accumulation of cancer stem cells in a time-dependent manner. Overall, we present an attractive, cost-effective method of culturing cells under appropriate physoxic or hypoxic conditions that is easily implementable in any wet laboratory equipped with cell culture tools.

## 1. Introduction

**Figure 1:**
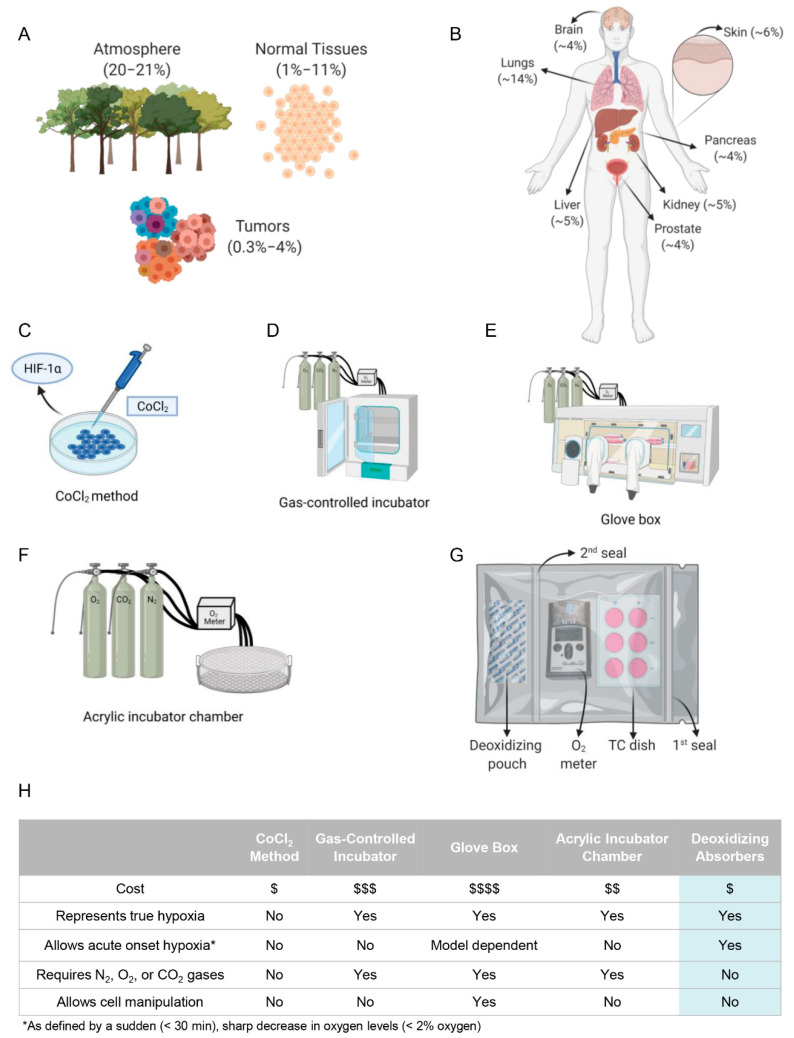
Available methodology to recapitulate in vitro oxygen levels found in the atmosphere, normal tissues, and tumors. (**A**) The schematic representations illustrate the oxygen concentration of atmospheric oxygen concentration (20.8%), as well as healthy tissues (1–11%) and tumors (0.3–4%). (**B**) Physoxic concentrations vary among the body tissues. The different oxygen concentrations are shown in parenthesis. (**C**–**G**) Many methods have been used to induce hypoxia. Cobalt chloride (**C**) is a method to achieve a chemically induced pseudo-hypoxia, whereby HIF-1α is upregulated to mimic hypoxia without truly adjusting the oxygen concentration. Gas-controlled incubator (**D**), glove boxes (**E**), acrylic incubator chambers (**F**), and here the use of deoxidizing absorbers (**G**) represent current methodologies that utilize nitrogen and carbon dioxide gas to achieve hypoxia. (**H**) Each methodology for achieving hypoxia, including the one we present in this manuscript, has benefits and limitations as shown in the table.

The importance of oxygen has gained attention from many civilizations across time. This invisible gas used in respiration is essential for life as we know it today. The reduced concentration of oxygen, known as hypoxia, plays an important role in a myriad of biological mechanisms, ranging from healthy to pathological conditions such as cancer [1,2,3,4]. Specific oxygen concentrations play distinctive biological roles in cellular metabolism and low concentrations may be crucial to natural homeostasis, as physiological oxygen concentration ranges from 1% to 11% (Figure 1A). Interestingly, there is significant variation in physoxia across various tissues of the body (Figure 1B), with oxygen levels of approximately 12–15% in the lung airways (bronchi to alveoli) and reaching as low as 3.4% to 6.8% in the arterial blood supplying peripheral tissues [5,6,7,8].

Solid tumors are particularly susceptible to undergoing low oxygen concentrations during growth. When under hypoxic conditions, tumor cells accumulate HIF-1α, which impacts gene transcription leading to a poor prognosis [1,9,10,11]. The extent of hypoxia observed in cancerous tumors differs across the body, with head and neck cancers exhibiting a median oxygen concentration of 1.3–1.9% [12,13,14,15], while pancreatic tumors, for instance, present with an oxygen concentration of 0.3–1.2% [7,16,17,18]. The role of tissue-specific physoxia and the observed variation in tumor oxygenation throughout the human body bring about the question of how far we are from truly capturing human biology in vitro while using cell lines cultured at atmospheric levels of oxygen.

Current methods to recapitulate in vitro physoxic or hypoxic conditions are limited and range from lower cost chemical-induction of hypoxia-like effects to costly equipment. The use of cobalt chloride (CoCl_2_) is a common strategy to mimic hypoxia through multiple effects (Figure 1C), including binding to the PAS domain of HIF-1α to prevent pVHL binding and the subsequent ubiquitination and degradation of HIF-1α [19,20]. The administration of CoCl_2_, and to a lesser extent deferoxamine mesylate, is a fast and inexpensive technique capable of triggering an epithelial-mesenchymal transition (EMT) phenotype, with the accumulation of vimentin and fibronectin and increased cell polarity, all of which are consistent with hypoxia [21,22]. Although exciting, the molecular implications of chemical-induced hypoxia and the lack of actual changes in the oxygen concentration constitute serious limitation factors [22,23,24]. The use of laboratory equipment capable of inducing hypoxia through nitrogen injection better recapitulates the physiological conditions of hypoxia. As shown in Figure 1D,F, these strategies rely on a nitrogen injection mediated by a gas-controlled incubator or a glove box device [25]. The advantage of these systems is the avoidance of chemicals that can alter cell behavior, as seen with cobalt chloride treatment [22,23,24].

Nonetheless, the cost of implementing nitrogen-based strategies is high, and sustaining a constant hypoxic oxygen concentration is difficult [26,27]. Here, we present a cost-effective alternative to culturing cells under hypoxic and physoxic conditions by controlling the precise oxygen concentration with an oxygen absorber containing active powdered iron oxide (Figure 1G). The in vitro study of different tissues presenting distinct physoxic levels and hypoxic conditions is challenging and costly. Some of the advantages and disadvantages are summarized in Figure 1H. We further demonstrate that the iron oxide deoxidizing absorbers effectively trigger the cellular phenotype of tumor cells, including the acquisition of an EMT phenotype, cell cycle arrest, and the activation of tumorigenic pathways observed in hypoxic tumors.

## 2. Material and Methods

### 2.1. Hypoxia-Induced Deoxidizing Absorber Method and Cell Lines Culture Conditions

**Figure 2:**
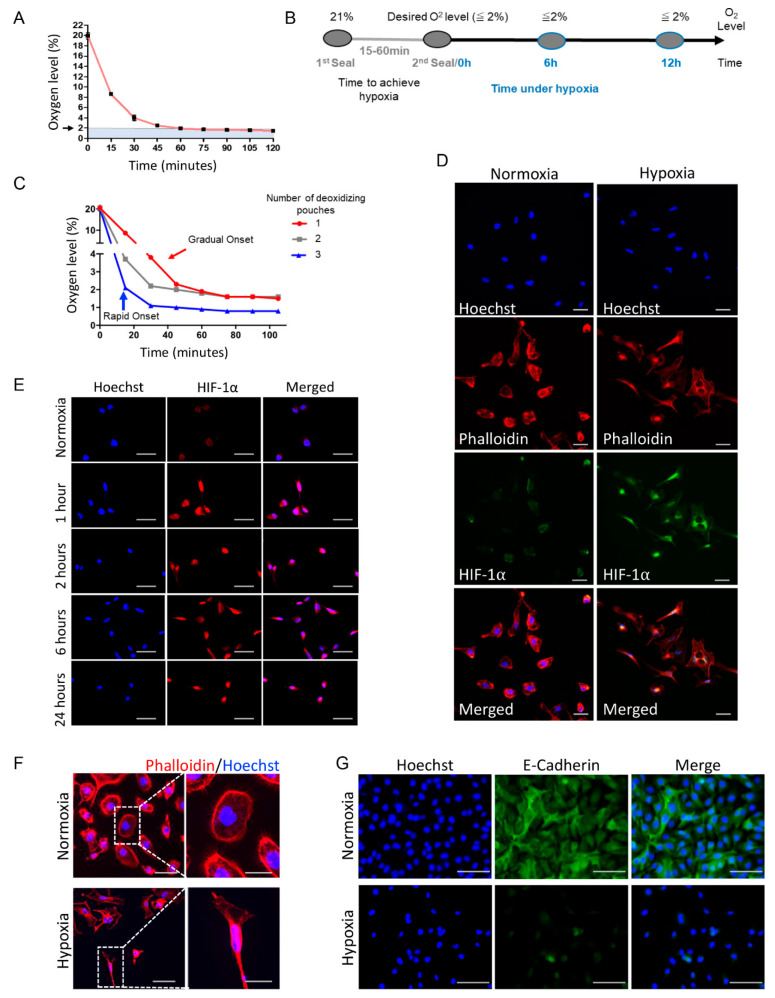
Implementation of deoxidizing absorbers to induce hypoxia and changes in cellular physiology. (**A**) The graphic depicts the deoxidizing absorber’s ability to reduce oxygen concentrations leading to hypoxic levels (<2%). (**B**) The diagram illustrates the timeline of the experiments. The time to hypoxia varies from minutes to an hour, depending on the number of oxygen absorbers used. After the desired oxygen concentration is achieved, the chamber receives a second seal that isolates the oxygen absorbers from the hypoxic chamber. Hypoxia or specific low oxygen levels can be maintained as constant over time. (**C**) The use of one deoxidizing absorber enabled a gradual onset of hypoxia (red line). Note that ≤2% oxygen concentration was achieved in 1 h. In contrast, multiple oxygen absorbers allowed for a sharper, more rapid onset, resulting in ≤2% oxygen concentration in 15 min (blue line). (**D**) Immunofluorescence staining for phalloidin (red) reveals spindle-shaped or fusiform morphological change denoting activation of an EMT phenotype under hypoxia compared with epithelioid shape cells from normoxia group. Increased levels of HIF-1α (green) are observed in hypoxic conditions compared with normoxia levels. Counterstaining is shown in blue (Hoechst 33342) (scale bar 50 µm). Cells were maintained under hypoxia for 18 h. (**E**) Immunofluorescence staining of the time-course assay for Hif-1α demonstrates increased protein levels after 1 h of hypoxia and sustainment of Hif-1α levels up to 24 h (scale bar 50 µm). (**F**) Immunofluorescence for phalloidin demonstrates hypoxia-induced activation of an EMT-like phenotype in cells compared with epithelioid shape cells cultured under normoxia (scale bar 50 µm, inserts 25 µm). (**G**) Immunofluorescence staining for the epithelial marker E-cadherin (green) depicts the loss of expression upon cell culture under hypoxic conditions (scale bar 100 µm).

Hypoxic culture conditions were achieved using 2000 cc oxygen absorbers containing active powdered iron oxide (BayTec Containers, Bacliff, TX, USA) placed in a self-sealed plastic bag containing the cell culture dishes. Oxygen levels were constantly monitored using an oxygen meter (Pro Gas Badge, Grainger, Lake Forest, IL, USA). Once the required level of O_2_ was achieved, a second seal was placed in the bag to isolate the cells. The oxygen meter from the oxygen absorbers is referred to as the hypoxia-induced deoxidizing chamber (Figure 2A). Four cell lines were used, including two head and neck squamous cell carcinomas (HNSCC) (WSU-HN12 and WSU-HN13), one human immortalized skin cell line (HaCaT), and one adenoid cystic carcinoma cell line from the salivary glands (UM-HACC-2A), all cultured in normoxia or under hypoxia. WSU-HN12, WSU-HN13, and HaCaT cell lines were cultured using DMEM (HyClone Laboratories, UT, USA) supplemented with 10% fetal bovine serum (FBS) (HyClone Laboratories, UT, USA) and 1% antibiotic-antimycotic (Thermo Fisher Scientific, Waltham, MA, USA) at 37 °C in a 5% CO_2_ humidified environment. The UM-HACC-2A cell line was cultured using DMEM (HyClone Laboratories, UT, USA), supplemented with L-glutamine (Gibco, Waltham, MA, USA), 1% antibiotic-antimycotic (Thermo Fisher Scientific, Waltham, MA, USA), 10% FBS, (HyClone Laboratories, UT, USA), 2% Bovine Brain Extract (Lonza, Walkersville, MD, USA), 20 ng/mL rhEGF (PeproTech, Cranbury, NJ, USA), 0.4 µg/mL human hydrocortisone (StemCell Technologies, Vancouver, BC, Canada), 5 µg/mL human insulin (Sigma-Aldrich, St. Louis, MO, USA), and 1% amphotericin B (Sigma-Aldrich, St. Louis, MO, USA). Cells were cultured under hypoxia for 1, 2, 6, and 24 h after achieving oxygen levels of 2%. Cell identity confirmation was carried out using STR (short tandem repeats) profiles.

### 2.2. Immunofluorescence Assay

Cells were seeded onto glass coverslips previously placed into tissue culture (TC) wells. Cells were cultured under the hypoxic conditions described above. Cells were fixated using 3% paraformaldehyde for 15 min at room temperature and washed three times using phosphatase-buffered saline solution (PBS), followed by the addition of blocking buffer (3% (*w*/*v*) bovine serum albumin (BSA) containing 0.5% (*v*/*v*) Triton x-100 in PBS. The primary antibodies were diluted in blocking buffer and incubated overnight. Rhodamine phalloidin (1:50; Cytoskeleton, Inc., Denver, CO, USA), anti-HIF-1α (1:800; Cell Signaling Technology, Danvers, MA, USA), anti-Phospho-S6 (1:100; Cell Signaling Technology), anti-Snail (1:100; Cell Signaling Technology), anti-Twist (1:50; Abcam), and anti-E-cadherin (1:100; Cell Signaling Technology) were used along with Alexa Fluor secondary antibodies (Alexa 568, Thermo Fisher Scientific). The cellular DNA material was stained using Hoechst 33342. Images were acquired on an ImageXpress Micro 4 system equipped with MetaXpress 6 software (Molecular Devices, San Jose, CA, USA) and using an ExiAqua monochrome digital camera attached to a Nikon E80i microscope and equipped with Nikon Elements NIS software (Nikon, Melville, NY, USA).

### 2.3. Reactive Oxygen Species and Superoxide Dismutase Assay

In order to monitor the endogenous levels of ROS and SOD in cells cultured under hypoxic and normoxia, we used the ROS-ID Total ROS/Superoxide detection kit (Enzo Life Sciences, East Farmingdale, NY, USA). Briefly, the oxidative stress and superoxide kit reagents (hydrogen peroxide, peroxynitrite, hydroxyl radical, NO, peroxyl radicals, and O_2_^−^) were reconstituted in anhydrous DMF to produce a final stock solution of 5 mM and stored at −20 °C, as directed by the manufacturer. Adherent HNSCC cells were cultured under hypoxic conditions (2% O_2_) for 24 h. The cells were processed as indicated by the manufacturer before they were analyzed using an Accuri C6 Plus flow cytometer and software (BD Biosciences).

### 2.4. Invasion Assay and Cell Cycle Analysis

Cells were seeded in 24-well plates over Millicell cell culture inserts (MilliporeSigma) pre-coated with a thin layer of fibronectin (BD Biosciences, San Jose, CA, USA). The upper side of the inserts received DMEM supplemented with 2% FBS, and the well plate received DMEM supplemented with 10% FBS. Cells from the control group were maintained under normoxia, while those of the hypoxia group were exposed to deoxidizing absorbers and maintained under <2% of oxygen for 24 h of cellular invasion. Residual cells located at the upper portion of each Millicell membrane were removed using a cotton swab, and invading cells were stained with hematoxylin and eosin (H&E). The total number of cells located in the lower chamber (invasive cells) was quantified. Images were taken using a color camera (Micropublisher 5.0; QImaging, Surrey, BC, Canada) attached to a Nikon Eclipse 80i microscope (Nikon). The images were analyzed using the ImageJ program (NIH). The cell cycle was performed using propidium iodide (PI) staining. Briefly, cells were cultivated under hypoxia versus normoxia for 24 h and harvested following fixation with 70% ethanol on ice for 2 h. The cells were resuspended in 0.5 mL PBS containing 0.25% Triton X-100 for permeabilization for 15 min on ice. Subsequently, cells were incubated with PBS containing PI (20 µg/mL) and RNase solution (10 µg/mL) (Sigma-Aldrich, St. Louis, MO, USA) for 30 min at room temperature. The relative number of cells in each phase of the cell cycle (G1, S, and G2/M) was accessed by flow cytometry (Accuri C6 Plus, BD Biosciences).

### 2.5. Identification of Head and Neck Cancer Stem Cells

Cancer stem cell (CSC) content was assessed during normoxia and hypoxia using the ALDEFLUOR™ Kit for ALDH Assays (Stemcell Technologies, Durham, NC, USA) in combination with a CD44 antibody conjugated with allophycocyanin (APC), as previously reported [28,29,30,31]. Briefly, tumor cells were cultured for 12 and 24 h under hypoxic conditions and were trypsinized and resuspended in FACS buffer containing CD44/APC antibody for 45 min. Following 3 washes with PBS, cells were incubated with ALDH reagents following the manufacturer’s protocol. The stained single-cell suspension was then analyzed by flow cytometry for double staining for ALDH and CD44.

### 2.6. Statistical Analysis

The statistical analyses were performed using GraphPad Prism 7 (GraphPad Software, San Diego, CA, USA). The statistical tests used were a one-way analysis of variance (ANOVA) and a student’s *t*-test. Data are expressed as mean ± SEM. Asterisks denote statistical significance (* *p* ≤ 0.05; ** *p* ≤ 0.01; *** *p* ≤ 0.001; **** *p* ≤ 0.0001, and ns *p* > 0.05).

## 3. Results and Discussion

### 3.1. Deoxidizing Absorbers Hypoxia Method Is Capable of Simulating Acute or Chronic Hypoxia

Much is known about the hypoxia-driven pathological events in cancer biology; however, little attention has been given to the low O_2_ gas concentration in maintaining health conditions. Oxygen concentrations are generally categorized as atmospheric oxygen concentration or normoxia, tissue oxygen concentration or physoxia, and reduced physiologic oxygen concentration or hypoxia. Interestingly, the physiological concentrations of oxygen (1–11%) are closer to hypoxia (<2%) than to the atmospheric oxygen concentration (~21%), which has implications for most investigations using cell culture nowadays, considering that the atmospheric concentration is widely misapplied to reflect physoxia [5,6,7]. The ability to accurately and precisely modulate oxygen concentration to reflect a particular physiological condition is important for hypoxia research. Slight differences in oxygen concentration can have significant biological implications. For instance, a study by Timpano et al. explored both normal and malignant cell lines, both of which presented with optimal growth under a distinct range in the oxygen concentration curve, which the authors cleverly named the “goldiloxygen zone”. Conversely, those cell lines underwent more damage, including DNA damage and decreased metabolism, at oxygen levels outside this range [5]. Consequently, many systems function within an optimal oxygen concentration and having an affordable technique that can produce in vitro situations more closely aligned with real physiology proves beneficial in oxygen studies.

In search of a new, cost-effective strategy for achieving hypoxia that would overcome many of the present methodologic issues, we focused on a hypoxia method that could utilize deoxidizing absorbers [32,33]. Intrigued by this underexplored technique, we further designed a deoxidizing absorber chamber with an oxygen absorber, cells, and an oxygen meter inside a double-sealed bag (Figure 1G). We first assessed its efficiency in decreasing O_2_ gas using one or multiple oxygen absorbers. Notably, we were able to achieve very low levels of oxygen (<2%) with the absorbers (Figure 2A,B). By using multiple deoxidizing absorbers, we increased the rate at which hypoxia was achieved. Furthermore, oxygen concentrations could be maintained at various times (Figure 2A–C). Ultimately, our technique allows for the modulation of the rate and duration of oxygen depletion in rapid-onset versus gradual-onset hypoxia and acute versus chronic hypoxia, respectively (Figure 2C). From a pathophysiological perspective, acute and chronic hypoxia are quite distinct, and both can be split into subtypes. Overall, acute hypoxia is characterized by severe oxygen reduction and can often be caused by mechanical obstruction. In contrast, chronic hypoxia is a long-standing oxygen reduction frequently observed in heavy smokers, primary and metastatic liver tumors, and other conditions [34,35,36,37,38,39,40]. Exposure to chronic hypoxia, when cells are placed under hypoxia for several hours or even weeks, is associated with a higher frequency of DNA breaks and less repair of DNA replication errors [37,41]. Acute hypoxia in tumors is known to cause genomic instability with p53-dependent apoptosis and DNA damage [37,41]. Furthermore, in a study by Cairns et al., it was shown that sarcoma tumors exposed to acute hypoxia presented a significant increase in lung metastases [42]. Acute and chronic hypoxia was also shown to decrease the radiosensitivity of gastric and esophageal cancer cells [43], reiterating the importance of considering the role of oxygen levels in cancer therapeutics. Ultimately, a typical tumor undergoes a cycling of oxygen concentrations, possessing chronic and acute hypoxia regions that are believed to significantly impact tumorigenesis and clinical responses to therapy [37].

### 3.2. Deoxidizing Absorbers as an Efficient Strategy to Control O_2_ Levels and Induce Accumulation of HIF-1α and Activation of EMT

About 25 years ago, the role of HIF-1α in hypoxia was identified [19,44]. Since then, HIF-1α has been related to a plethora of hypoxia-modulated mechanisms. Its effects gained special attention from cancer biologists who noticed an increased aggressive behavior associated with higher levels of HIF-1α, which became a target for cancer therapy [1,45,46]. Thus, we further assessed the levels of HIF-1α using the deoxidizing absorbers and observed the accumulation of HIF-1α in our cells (Figure 2D,E). With the hypoxic system, we demonstrated the accumulation of HIF-1α in the nucleus, contrasting this with the cells under normoxia. In the presence of oxygen, HIF-1α is destabilized by prolyl hydroxylation and lysyl acetylation within the oxygen-dependent degradation domain [47,48]. HIF-1α is then ubiquitinated by pVHL, leading to its degradation [1,11,47,48], which corresponds to the weak green staining of HIF-1α in the cytoplasm under normoxia (Figure 2D). The stabilization of HIF-1α is observed in cells under low oxygen levels, where it enters the nucleus and dimerizes with HIF-1β affecting transcription through an interaction with the hypoxia-response element regions (HREs) of the DNA [47,49]. We also observed that deoxidizing absorbers could sustain HIF-1α expression for up to 24 hours (Figure 2E). Along with the accumulation of HIF-1α, cells exposed to hypoxic conditions also underwent an EMT phenotype, as depicted by the phalloidin staining (Figure 2F).

Along with the accumulation of HIF-1α and the acquisition of an EMT phenotype, we also observed the loss of the epithelial cell marker E-cadherin under hypoxic conditions (Figure 2G). E-cadherin is an adherens junction protein responsible for the cell–cell contact of epithelial cells, and involved in intracellular organization through contact with the cellular cytoskeleton. The loss of E-cadherin is a hallmark of EMT [50].

### 3.3. Deoxidizing Absorbers Increase ROS Levels, mTOR Activation and Enhance Invasive Cell Behavior

**Figure 3:**
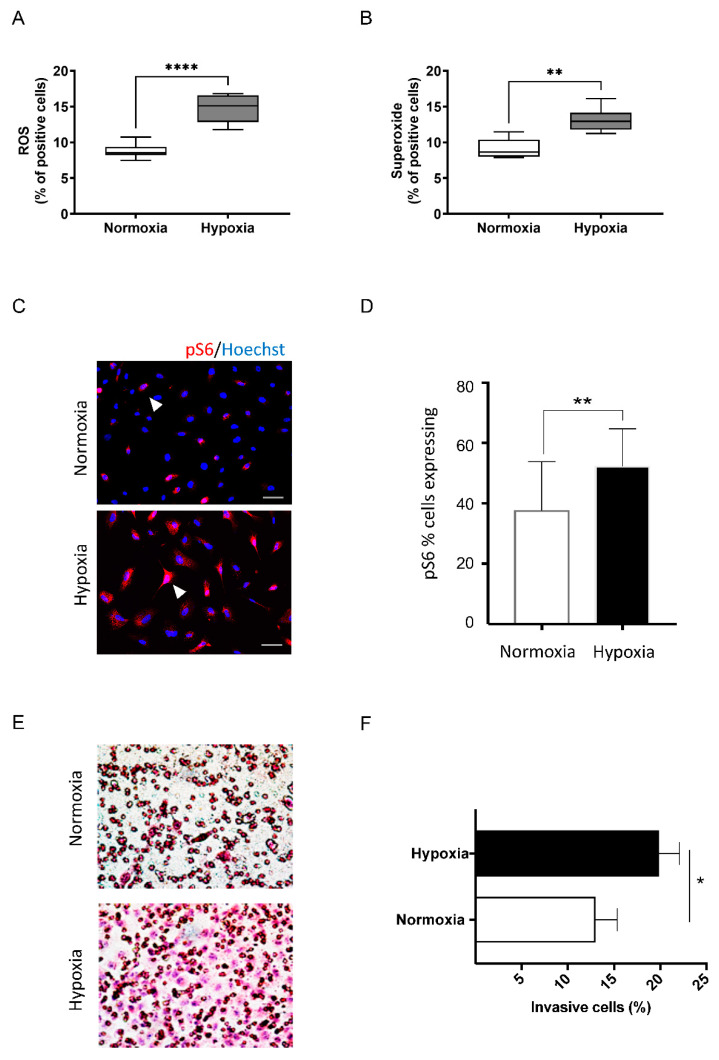
Cells in the deoxidizing absorbers chamber displayed increased levels of ROS, Superoxide, mTOR levels, and invasive capacity. Cells cultivated in the presence of deoxidizing display (**A**) increased ROS and (**B**) superoxide levels (**** *p* < 0.00001, ** *p* < 0.001, respectively). (**C**) Immunofluorescence demonstrates increased expression levels of the mTOR readout marker pS6 in cells cultured under hypoxia (scale bar 50 µm). (**D**) Quantification of positive cells for pS6 shows a statistically significant activation of mTOR signaling under hypoxia compared to the control (normoxia) (** *p*< 0.001). (**E**) Invasion assay using Millicell cell culture inserts during normoxia and hypoxia, demonstrating the enhanced invasive capacity of tumor cells cultured under hypoxic conditions. (**F**) Quantification of invading cells, demonstrating a statistically significant increase in invasive behavior in tumor cells under hypoxia (* *p*< 0.05).

Reactive oxygen species (ROS) is a term to designate reactive forms of oxygen, which have an essential biochemical role in life. Hypoxia generates ROS, which has been a target of study for cancer biologists particularly. Here, we have shown that head and neck cancer cells cultured under hypoxia using deoxidizing pouches present a spike in the intracellular levels of ROS and superoxide (**** *p* < 0.0001 and ** *p* < 0.01 respectively; Figure 3A,B). Solid tumors are known to contain hypoxic niches associated with increased ROS levels. Indeed, we have previously shown that hypoxic niches found in head and neck cancer supersede the protective ability of the tumor suppressor gene PTEN [51]. Apart from hypoxia, the loss of PTEN from HNSCC led to reduced levels of ROS, which suggests a mechanism for escaping apoptosis [51,52]. Our group observed that higher ROS levels presented a potential to break down the protein levels of PTEN [53]. Altogether, these findings point to a metabolic cycle that, in hypoxic conditions, tumor cells can generate cells capable of reducing ROS to evade apoptosis. Still, chronic tumor hypoxia leads to a ROS-resistance phenotype, which helps the cancer cells survive in reoxygenated areas; in other words, there are higher chances of successful metastasis, suggesting ROS-resistance as a promising target for cancer therapy [54]. The deoxidizing absorbers should be a useful strategy for understanding the metabolic pathway behind ROS modulation. With this in mind, we decided to verify the important cancer biology mTOR pathway, which is downstream of PTEN. To this end, we further analyzed the expression of pS6, a ribosomal protein, here serving as a readout or biomarker of the mTOR pathway. Under hypoxic conditions, pS6 levels accumulated in the head and neck cancer cells (Figure 3C,D, ** *p* < 0.01). Thus, the hypoxic condition caused by the deoxidizing absorber can activate the mTOR pathway. Along with the increased expression of mTOR signaling, we also observed the enhanced invasive capacity of tumor cells when cultured under hypoxia compared with normoxia conditions (Figure 3E,F) (* *p* < 0.05).

### 3.4. Hypoxia Mediated by Deoxidizing Absorbers Effectively Induces Cell Cycle Arrest, Accumulates Cancer Stem Cells, and Induction of SNAIL and Twist across Several Epithelial Cell Lines

**Figure 4:**
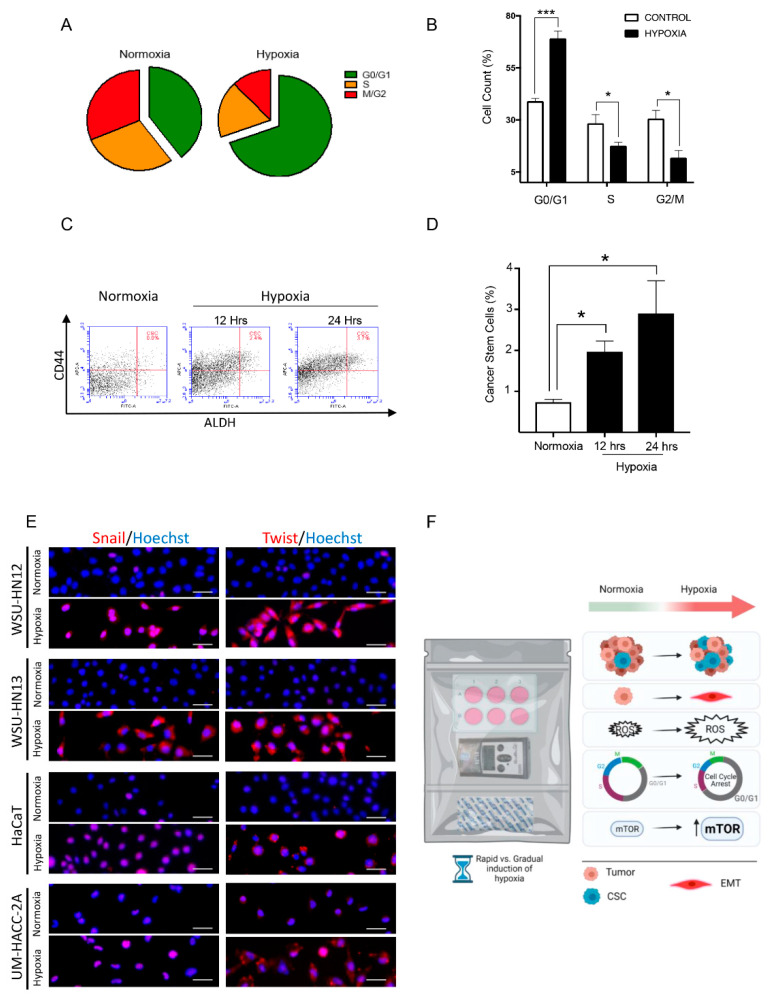
Deoxidizing absorbers method induces cell cycle arrest, accumulation of cancer stem cells, and accumulation of Snail and Twist. (**A**,**B**) The pie graphic demonstrates an increased number of cells in the G0/G1 phase under hypoxia compared to normoxia (*** *p* < 0.001). (**C**,**D**) Deoxidizing absorbers induced the continuous accumulation of cancer stem cells measured at baseline (normoxia), 12 h, and 24 h (ALDH^+^/CD44^++^ positive cells) (* *p* < 0.05). (**E**) Immunofluorescence staining for Snail and Twist on 4 different epithelial cell lines. WSU-HN12 and WSU-HN13 are squamous cell carcinomas, HaCaT is a human immortalized skin cell line, and UM-HACC-2A is a salivary gland adenoid cystic carcinoma cell line. Note the weak immunofluorescence staining for Snail (Alexa 568—red) in all 4 cell lines cultured under normoxia compared with hypoxic conditions (scale bar 50 µm). Immunofluorescence staining for Twist (Alexa 568—red). Note the weak staining in all 4 cell lines cultured under normoxia and accumulation of Twist in all cell lines during hypoxia. Hoechst 33,342 counterstaining for DNA content is in blue (scale bar 50 µm). (**F**) Diagram representing the biological effects of deoxidizing-absorbers-induced hypoxia over epithelial cells. Deoxidizing absorbers may be used to achieve rapid vs. gradual onset of hypoxia. They are also effective in triggering the EMT phenotype in epithelial cells, increasing the intracellular levels of ROS, activating the mTOR pathway, inducing cell cycle arrest (G0/G1), and in promoting the accumulation of cancer stem cells.

In many cells, cell cycle arrest is modulated in a HIF-1α dependent manner [55,56,57]. Concordant with this notion, the cells in the deoxidizing chamber revealed an increase in cells in the G0/G1 phase, suggesting a G0/G1 cell cycle arrest (Figure 4A,B, * *p* < 0.05, *** *p* < 0.001). The accumulation of quiescent-like cells prompted us to explore the potential effects of hypoxia modulated by this method on the levels of cancer stem cell markers. According to a study by Mathieu et al., the expression profiles of eleven cancer cell lines (from prostate, brain, kidney, cervix, lung, colon, liver, and breast tumors) grown under hypoxic conditions shared an overlapping gene expression signature with nine human embryonic stem cell (hESC) lines [58]. Furthermore, Mathieu et al. noted a correlation between hESC marker expression and stem cell character in hypoxic gliomas, associating HIF with increased stemness [58]. Supported by these results, we analyzed the percentage of CSC in head and neck cancers cultured in the deoxidizing chamber. An increase in the number of stem cells was observed (Figure 4C,D, * *p* < 0.05). Consequently, given the fact that the number of cancer stem cells is very low compared to the cells forming the tumor bulk [59,60,61], our results support a prospective use of this technology to rapidly boost this population of cells and enhance studies that explore the biology of cancer stem cells. Our previous data demonstrate an enhanced polarization of phalloidin filaments upon exposure of cells to deoxidizing absorbers, along with the loss of the epithelial cell marker E-cadherin and increased cellular invasion which strongly suggests that our system can efficiently trigger EMT. To confirm that EMT is consistently induced using deoxidizing absorbers, we decided to test our hypoxia method on different cell lines of epithelial origin. We also decided to use Snail and Twist as two well-defined molecular markers for EMT. We used two squamous cell carcinoma cell lines, one human immortalized skin cell line, and one salivary gland tumor cell line exposed to deoxidizing-absorber-induced hypoxia and immunostained for Snail and Twist. Our data showed that all cell lines exposed to deoxidizing absorbers presented enhanced expression of both EMT markers after 24 h of hypoxia (Figure 4E). In all, we have successfully demonstrated that hypoxia induced by oxygen depletion using deoxidizing absorbers is a viable, cost-effective, and user-friendly strategy to study several aspects of tumor and cellular biology, including the accumulation of cancer stem cells, activation of EMT, accumulation of intracellular ROS levels, induction of cell cycle arrest, and activation of mTOR signaling (Figure 4F).

## 4. Conclusions

The deoxidizing absorbers hypoxia method allows researchers to modulate the oxygen concentration to more accurately reflect hypoxia, physoxia, or normoxia. Notably, the use of this technique facilitates the acquisition of acute and chronic hypoxia, which are difficult to achieve with current methodologies using a cost-effective method. The validity of this technique was demonstrated through its ability to replicate routine hypoxic events, including the accumulation of HIF-1α, the expression of an EMT phenotype, increased levels of ROS and the activation of the mTOR pathway as evidenced through pS6 expression, as well as the induction of cell cycle arrest and CSC accumulation (Figure 4F). While conventional methodology requires chemical (i.e., CoCl_2_) or costly nitrogen-induced hypoxia, this technology opens the door for developing new research strategies that aim to resemble the levels of O_2_ present in in vivo biology.

## Data Availability

The data generated in the present study may be requested from the corresponding author.
